# Identifying the Knowledge Structure and Trends of Outreach in Public Health Care: A Text Network Analysis and Topic Modeling

**DOI:** 10.3390/ijerph18179309

**Published:** 2021-09-03

**Authors:** Sooyeon Park, Jinkyung Park

**Affiliations:** 1 College of Nursing, Korea University, Seoul 02841, Korea; isypark@korea.ac.kr; 2 College of Nursing, Chonnam National University, Gwangju 61469, Korea

**Keywords:** outreach, public health care, text network analysis, semantics, topic modeling

## Abstract

Outreach programs are considered a key strategy for providing services to underserved populations and play a central role in delivering health-care services. To address this challenge, knowledge relevant to global health outreach programs has recently been expanded. The aims of this study were to analyze the knowledge structure and understand the trends in aspects over time and across regions using text network analysis with NetMiner 4.0. Data analysis by frequency, time and region showed that the central keywords such as patient, care, service and community were found to be highly related to the area, target population, purpose and type of services within the knowledge structure of outreach. As a result of performing topic modeling, knowledge structure in this area consisted of five topics: patient-centered care, HIV care continuum, services related to a specific disease, community-based health-care services and research and education on health programs. Our results newly identified that patient-centered care, specific disease and population have been growing more crucial for all times and countries by the examination of major trends in health-care related outreach research. These findings help health professionals, researchers and policymakers in nursing and public health fields in understanding and developing health-care-related outreach practices and suggest future research direction.

## 1. Introduction

Over the last few decades, health disparities have remained a major public health concern worldwide. Disparities in health care cause social problems (e.g., regional disparity of life expectancy) that result in excessive health-care costs [1]. Recently, there has been a sharp rise in the number of global health outreach programs to address this challenge in the disadvantaged communities [2,3]. Generally, vulnerable populations are exposed to health risk factors and reported to have high incidences of chronic and transmitted diseases [4]. Consequently, understanding how health-care disparities occur in the health-care delivery system and how they can be eliminated remains a paramount and universal pursuit [1].

To reduce health disparities, outreach programs are considered a key strategy for providing services to underserved or hard-to-reach groups and play a central role in delivering health-care services to them [5]. In fact, among other available approaches, outreach services can enhance access to health workers and improve overall retention even at the country level [6], and previous studies have reported their effectiveness in addressing disparities through customized interventions [7]. Further, community health-care professionals have frequent contact with at-risk populations and play a pivotal role in servicing them, not only by providing treatment and medical resources but also through education. Therefore, health-care professionals’ knowledge about outreach and their effective management skills are important factors for determining the quality of outreach services.

With a growing interest in outreach, researchers have consistently conducted quantitative and qualitative research on the topic. There has also been an increasing demand for outreach research reviews. However, although extensive outreach research has been conducted in nursing, public health and other fields, reaching an agreement on the clear definition of outreach remains a challenge [8]. Since the inception of the outreach program services for underserved communities several decades ago, the attributes of outreach and the role of the nurse and community team [9,10] has been continuously discussed. A previous study reported that outreach is one of the functions of public health nurses and particularly it is useful for meeting the needs of vulnerable populations, including high-risk groups [11]. Additionally, outreach is one of the interventions of the public health intervention wheel model as a framework for understanding PHNs’ practices [12]. Therefore, to better understand and broaden knowledge about outreach, it is necessary to review the research on it. Although meta-analysis and systematic reviews using secondary data as an accumulation of outreach research are also increasing, there are some limitations to such studies; their strict methodology tends to focus on a specific topic or knowledge of a few experts, thus failing to cover the studies comprehensively [13,14].

To establish the accumulated knowledge within a study field, the contents of previous research on that discipline need to be quantitatively analyzed, ensuring that various research topics are covered. There are extensive extant outreach-related studies; therefore, to sort them and understand outreach better, it is necessary to identify their main topics. Social network analysis (SNA) is an analytic and predictable method for extensive amounts of data and is used to examine the contextual meanings of words and their relationships. Texts can be coded and analyzed as networks of concepts referred to as maps or semantic networks [15]. Text network analysis (TNA) is useful for analyzing a wide range of text materials and topics in big data, using computer programs for SNA; it has recently been used in many disciplines, including nursing [16,17]. TNA enables the identification of knowledge structures and research trends. Knowledge structure analysis using text networks quantitatively derives key concepts in a particular field and visualizes relationships between key concepts [18]. Using the keyword frequency and co-occurrence search features, text network analysis enables researchers to identify the influence of words and research trends [19,20]. Although numerous outreach studies have been conducted in various fields, to the best of our knowledge, outreach remains largely unexplored in health care, and thus requires examination.

Such an examination should be preceded by a clear grasp of outreach research topics in public health settings in order to present appropriate future outreach research directions. Knowledge structures discovered through text network analysis studies help view current research trends systematically and identify contextual relationships between topics or research trends over time. Based on the resultant knowledge structure, we can reflect on the research trends and suggest future research directions [21]. Therefore, in this study, we utilized text network analysis to visualize relationships between key concepts in outreach studies, identify the outreach research trends over time and explore the features of the resultant knowledge structure on outreach in health care by country.

## 2. Materials and Methods

### 2.1. Study Design

This is a quantitative content study that utilizes text network analysis to explore keywords and research topics by constructing a network based on the keywords’ co-occurrence rate within selected outreach-related literature in health-care settings.

### 2.2. Research Procedure

Keyword network analysis assumes that a set of keywords representing the core contents can be extracted from literature [18]. Such representative data from keyword analysis may include title, abstract, author keyword and keyword data. In this study, we selected author keywords to identify the research attributes reflecting the researchers’ intentions. Our research process was as follows: (1) data collection of articles, (2) extraction of keywords and preprocessing, (3) generation of co-occurrence keywords matrix and network and (4) analysis of knowledge structure and visualization.

#### 2.2.1. Data Search and Collection

**Figure 1:**
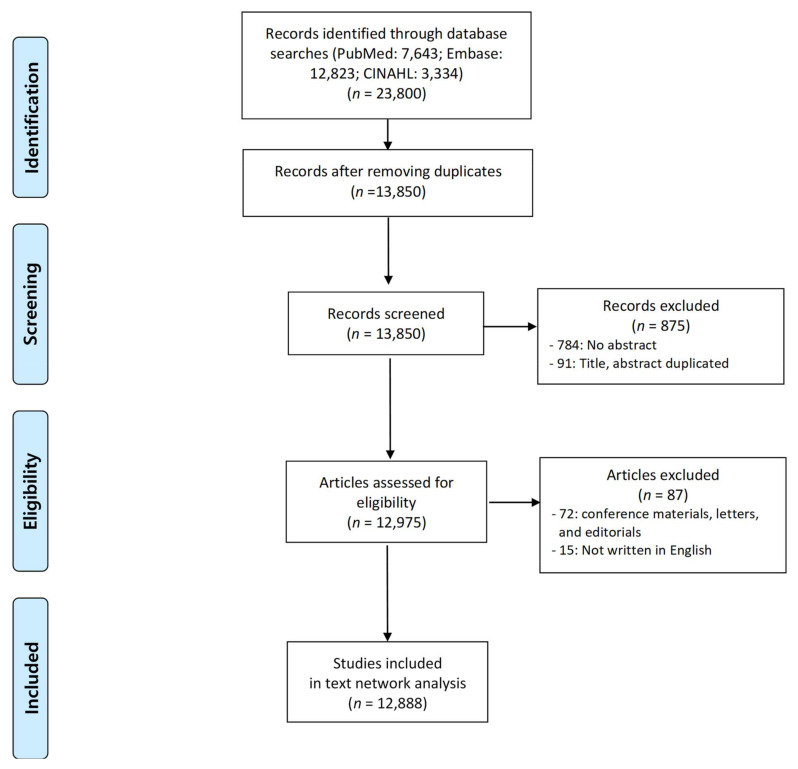
The flowchart of article inclusion process.

Research on public health-care outreach was collected from databases including PubMed, Embase and CINAHL (Cumulative Index to Nursing and Allied Health Literature). We searched the sources using outreach- and health-care-related terms in the title and abstract fields, limiting the articles to those published up until 2020 and written in English (Table S1). In total, 23,800 studies were identified. Of these, 12,888 studies were retrieved after excluding duplicates or articles without an abstract. The article inclusion process is summarized in Figure 1. We identified vital information from these 12,888 studies using citation information in databases and the region information of each study was defined from additional sources such as author and abstract information. A predefined excel file was used to organize the information (Table S2).

#### 2.2.2. Keyword Extraction and Preprocessing

We organized each study into one row by ID number, author, journal, year of publication and abstract to extract keywords. We varied the keywords and, before generating the keywords matrix, refined them to select those that were meaningful from the text. To refine the words, the researchers developed dictionaries comprising thesaurus, defined words and exclusion words. First, we unified words and abbreviations with the same or similar meaning and designated them a single representative word. In addition, to prevent overlapping uses of the same meaning during analysis, we processed the words by converting upper cases to lower cases and changing plural forms to singular ones. Second, two or more morphemes were grouped and specified to be extracted as a single word. Third, an exclusion list was developed by determining the morphemes to be excluded from the analysis, such as analytic terms and abstract forms. During the dictionary generation process, the researchers repeated the words analysis in the abstract and finally agreed on the words to be registered in the dictionary through discussion.

#### 2.2.3. Generation of Keyword Matrix and Network

After applying the thesaurus, defined words and exception word dictionary to the NetMiner version 4.4.3. (Cyram Inc., Seongnam, Korea), 48,931 keywords were identified along with their appearance frequencies. During text network analysis, the main phenomena are more clearly identified by focusing on repetitive subject words; generally, only keywords whose appearance exceeds a certain frequency are included in the analysis [22].

In the analysis, node means the subject words of each paper, whereas the scope of co-occurrence refers to one sentence containing the subject words of the paper. Here, when the same subject words emerged simultaneously in different papers, links were constructed and a network was formed. In this work, we created a matrix that valued the co-occurrence frequencies between previously selected keywords and built a network of subject keywords that represented co-occurrence relationships. Two words frequently appearing together were considered as having similar associations and important contextual relationships [19]. Further, we generated 922,012 one-mode matrices and analyzed the studies at 10-year intervals to identify changes in the outreach research subject over time. We then identified each study’s country of publication to explore the knowledge structure features of health-care outreach by region.

#### 2.2.4. Keyword Analysis and Visualization

We analyzed keyword centrality using the developed matrix and selected major keywords within the network to identify the knowledge structure of the research. Centrality is an indicator of how many nodes are present in a network, evaluated based on their relative ranking; keywords with high centrality are considered key keywords. Here, we analyzed three centrality indicators mainly used in text network analysis: degree centrality, closeness centrality and betweenness centrality [23,24]. Degree centrality measures how many links the nodes have in a network and evaluates the co-occurrences of keywords. Closeness (or proximity) centrality is to measure one node to the other nodes’ sum distances and to show how close one node is to another [25]. Betweenness centrality is to measure one node undertaking a “mediation” role in a network, and the extent to which one node acts as a bridge connecting other nodes in building a network [25].

To simplify the visualization of the network structure, the nodes and connectivity strengths to be included in the sociogram were determined and major keywords with an occurrence frequency of at least 30 values were selected in the filtering process. The network data and their analysis results were visualized through graphical presentations using the NetMiner version 4.4.3.b program.

#### 2.2.5. Topic Modeling

Since there is a huge amount of data available on any specific topic, topic modeling was performed to understand the topic of the research field. It is an unsupervised natural language processing method that analyzes non-numeric data such as text data in abundance, and aggregates and understands those data making them interpretable to interested audiences [26]. For our topic modeling, we performed Latent Dirichlet Allocation (LDA) analysis, whose algorithm is the most popular and frequently used among other topic modeling methods [27,28,29]. The algorithm identifies hidden topics in documents, topics of entire document sets and topic ratios for each document, as well as calculates the probability that each word will be included in each topic [30]. It is challenging to select the optimal number of topics in LDA modeling [29]. We conducted analysis on a number of topics with alpha 0.1 and beta 0.01 using the standard method of Bayesian statistics [29,31]. Additionally, we applied a time-interval-based evaluation to identify the changes in research topics over time.

## 3. Results

### 3.1. Keywords and Knowledge Structure of Health-Care Outreach Research

**Figure 2:**
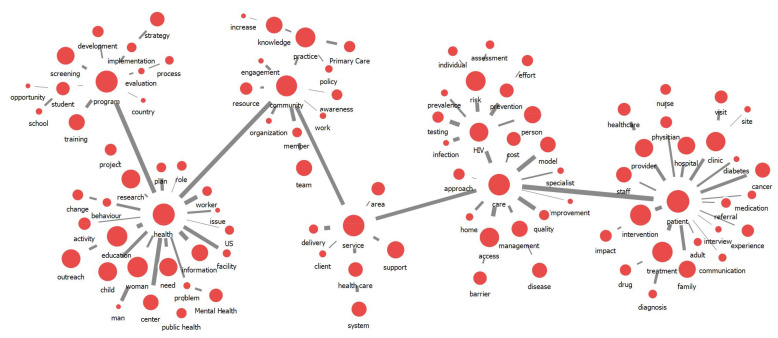
Overall knowledge structure for outreach research. The size of the node indicates the degree centrality of the keyword, and the width of the line indicates the strength of the link between keywords.

Table 1 presents the top 30 keywords by frequency, degree centrality and betweenness centrality indices, which were calculated from the extracted main words. These keywords also represent the highest centrality of direct connectivity to other keywords. Regarding the knowledge structure of outreach research, we found that six central keywords—“patient”, “care”, “service”, “community”, “health” and “program”—were highly related to other keywords (Figure 2). Considering the centrality degree, each colony was created around these six keywords. The six keywords appeared to play central roles in the network and served as interchanges and bridges.

### 3.2. Trends in Health-Care Outreach Research over Time

In total, 12,888 articles on health-care-related outreach were published over the 46-year period of 1975–2020. The number of articles by year is shown in Figure S1. We analyzed trends in the research by 10-year intervals. Throughout the 46 years, the keywords “programs”, “services”, “health”, “community” and “care” continued to rank among the top six. From 1975 to 1990 (phase I), “hospital”, “family” and “immunization” emerged as keywords compared to during other periods. From 1991 to 2000 (phase II), “patient” began ranking as the top keyword and “HIV” emerged and maintained a position as a significant centrality keyword. From 2001 to 2010 (phase III), keywords such as “management” and “team” emerged, and “service” became the most significant centrality keyword. The 2011–2020 period (phase IV) featured the emergence of keywords such as “provider,” “training,” and “support” while “patient” became the most significant centrality keyword (Table 2).

### 3.3. Trends in Health-Care Outreach Research by Region

**Figure 3:**
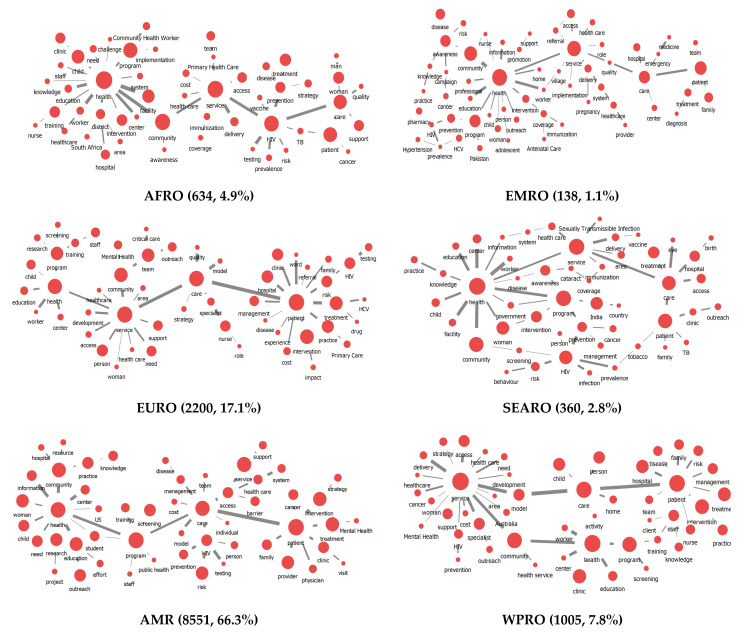
Knowledge structure of outreach research by each region. The size of the node indicates the degree centrality of the keyword, and the width of the line indicates the strength of the link between keywords; AFRO = African Region; AMR = Region of the Americas, SEARO = South-East Asia Region; EURO = European Region; EMRO = Eastern Mediterranean Region; WPRO = Western Pacific Region.

**Figure 4:**
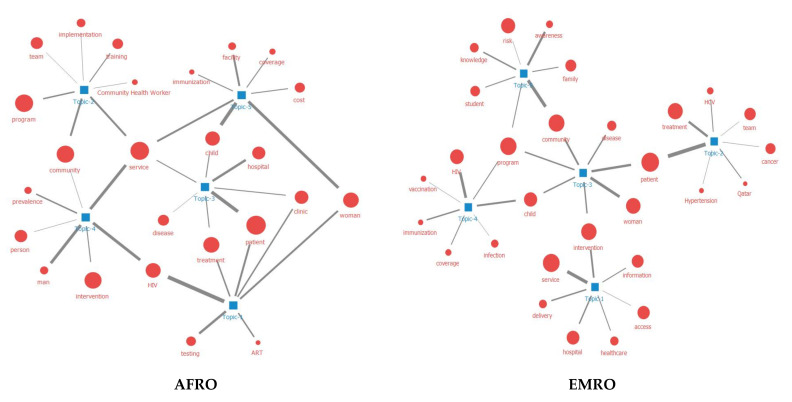

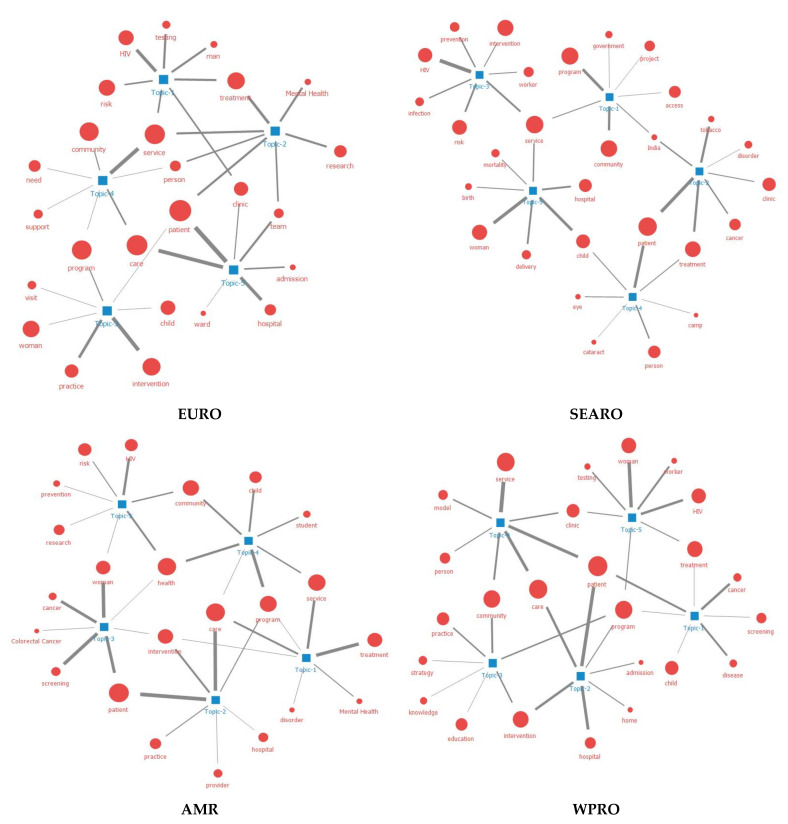
Topic modeling of outreach research by each region. The size of the node indicates the degree centrality of the keyword, and the width of the line indicates the strength of the link between keywords; AFRO = African Region; AMR = Region of the Americas, SEARO = South-East Asia Region; EURO = European Region; EMRO = Eastern Mediterranean Region; WPRO = Western Pacific Region.

We divided the world into six regions according to the WHO classification method [32] and derived research trends for each region (Figure 3). Additionally, we identified the top 30 keywords by degree centrality for each region (Table 3). As shown in Figure 4 and Table 4, “HIV” was the most significant centrality keyword in the Africa region (AFR) than any other region and keywords such as “district” and “TB (tuberculosis)” emerged in the African Region (AFR). In the Region of the Americas (AMR), which accounts for 66.3% of the literature, we derived results similar to the trend found in the general outreach research. The keyword “education” showed higher centrality, and “barrier” emerged as a keyword across the regions. In the South-East Asia Region (SEAR), unlike other regions, “India” and “government” appeared as keywords with high centrality. The European Region (EUR) accounted for 17.1% of the total literature, and in that region, the keyword “nurse” appeared with high centrality compared to in other regions. Additionally, “Mental health” and “Primary care” emerged as keywords with high centrality. In the Eastern Mediterranean Region (EMR), unlike other regions, keywords such as “HCV (hepatitis C virus)”, “coverage”, “pharmacy”, “Pakistan”, “referral” and “hypertension” emerged. In the Western Pacific Region (WPR), “model” and “home” emerged as high centrality keywords compared to in other regions.

### 3.4. Topic Modeling of Outreach Research

As previously mentioned, LDA topic analysis identifies topics commonly included in literature based on unsupervised learning. The topics are formed into keyword combinations based on statistics, then experts in the relevant field judge the meaning of the combinations using the statistics and derive meaningful topics. Here, seven rounds of LDA were performed on varying numbers of topics (K = 2, 3, 4, 5, 6, 8, 10). In the case of K = 2 and 3, it was difficult to derive meaningful content because they included too few subtopics. Since K = 10 included a large number of subtopics, there was a problem of overlapping topics. After the subtopics were grouped by the researchers through discussion, K = 5 topics with no overlapping meanings between groups were finally identified (Table 4). Each topic was ranked with reference to word weight, and the top 10 collocates in the corresponding topic were extracted. Weight is a value that represents the strength of each topic in the texts collected; it was expressed through a range of 0–1, where 1 indicated the most weighted. We combined meaningful keywords to form topic groups and derived five such groups as listed in Table 4. This process was similar to content analysis. The network between keywords in each topic group is shown in Figure 4.

## 4. Discussion

This study aimed to provide insights into health-care-related outreach research by investigating the main keywords published until the year 2020 through text network analysis. The study also investigated global research trends by period and geographical region. Specifically, we quantitatively analyzed 12,888 research papers published over a 46-year period and discovered the meaning of outreach in this era. Here, we present a scientific perspective on the subject attained by observing the global trends of core outreach-related research topics and identifying the outreach knowledge structure. Focusing on the six previously identified central keywords, we sought to identify the central concept of outreach related research. Outreach research, shown through the macro network analysis, indicated that patient-centered health-care services are being provided through community-based outreach programs.

We analyzed the research trends by decade and found an approximate six-times increase in the number of research topics since 2010. As part of the Patient Protection and Affordable Care Act (ACA) enacted in March 2010, financial support has been provided to implement ACA outreach and education efforts in rural areas through the health-care outreach grant program [33]. In 2011, WHO became aware of the primary health-care worker shortage in rural and remote areas, and to solve the causal staff retention problem, the organization reported the initiation of an accessibility improvement program through international calls for action [6]. In the same year, South Africa implemented a plan to re-establish the health-care system, which included outreach teams consisting of national community health workers (CHW) [34]. These global changes could have invoked the necessity of outreach research and presented the opportunity to expand the extant literature.

In Phase I (prior to 1990), the keywords “hospital”, “family” and “immunization” emerged and we found that health topics within infectious disease prevention research during this period were related to these keywords. The Expanded Program on Immunization (EPI) was aimed at ensuring that the morbidity rates of various communicable diseases are reduced by 1990. This immunization was implemented as an outreach service targeting families, including children and women, and as part of general health care at hospitals and primary health centers [35].

In Phase II (1990s), the keyword “HIV” emerged. This is because the HIV epidemic had been increasing since its onset 20 years prior to 1990 and was reported to be the fourth biggest cause of death worldwide [36], and the HIV preventive recommendation reflects the cumulative evidence from community-based research [37,38]. Community-based outreach has been regarded as an effective public health strategy to reach inaccessible and far-from-treatment populations, providing such residents with the means to change their behavior and reduce their HIV acquisition and transmission risks [39].

In Phase III (2000s), new keywords, “service”, “management” and “team” emerged. During this time, patient-centered care was emphasized and considered an integral part of teams collaborating with health professionals. Patient-centered health-care systems can provide high quality care and augment patient services [40]. Moreover, ward-based outreach teams (WBOTs) are increasingly important for primary health care globally [41]. WBOTs comprised of CHWs is one of the three components of the primary health care re-engineering strategy for improving health outcomes in South Africa such as prevention of HIV infection by health education, linkage to care and adherence support [42].

During Phase IV (2010s), the keywords “provider”, “training” and “support” emerged as keywords. According to previous study, CHWs in primary care were defined as providers of patient-facing support and primary care services via a training process and important role players in increasing primary access to health, quality and delivery [43]. These CHWs performed various functions—including health education, coaching, social support, literacy support and coordination—that promoted health outcomes in primary care settings [43].

Based on WHO’s six regional classification criteria, we identified regional global trends. “HIV”, “district” and “TB” were the keywords for the AFR. Africa, including the sub-Saharan area, is the most HIV-affected region—accounting for two thirds of the global HIV infections [44]—with over 34% of those infected also suffering from TB in 2016 [45]. HIV and TB form a lethal combination, each speeding the other’s progress. The effects of HIV/AIDS have prompted the reconstruction of the public health system, requiring a focus on community outreach services and the formalization of CHW programs via government. However, to make the CHW program a success, district and sub-district health structures in South Africa have struggled to provide adequate facility-based care [46].

Regarding research quantity, the region with the most published outreach papers was the AMR. The population of the Americas totaled 992.2 million (13.5% of the global populace) in 2015; however, the region produced the highest percentage (66.3%) of overall outreach research, with the US accounting for most of the publications [47]. In this region, the keywords were “education” and “barrier.” Health education is central to primary health care, which in turn is the primary means of achieving “Health for All.” Therefore, health education is a vital duty of the health sector and other community workers who take part in primary health care [48]. Gruca [49] conducted a study on community clinic patients and health-care team members in rural areas to identify important patient barriers to HBV (hepatitis B virus) care and found that community outreach strategies improved access to care while improved education and counseling prevented infectious diseases.

“India” and “government” were the prominent keywords of the SEAR. According to a recent WHO report on this region, its share of disease burden is higher compared to other regions. The report showed that over 800 million people in the SEAR live without full coverage of essential health services and emphasized the importance of primary health care in reinforcing health-care systems and urging governments to improve health equity [50]. India encountered the largest disease burden among the SEAR countries. The Indian health-care system needed to change toward reinforcing the primary health care and ensuring effective outreach services in primary care [41].

In the EUR, the keywords “nurse”, “mental health” and “primary care” showed relatively high centrality. This result is associated with critical care outreach teams (CCOT) and assertive outreach service (AOS). Critical care outreach is a system offering intensive care to at-risk patients with critical illnesses, and most UK centers have nurse-led outreach systems [51]. The AOS is a community-based health-care approach providing easily accessible services to hard-to-reach and severely disadvantaged persons, specifically those with mental health problems [52]. Previous research suggested that assertive community treatment services focusing on hard-to-reach patients with mental health problems facilitated links to primary care and identified significant clinical improvements in Europe [53].

For the EMR, several of the keywords we identified represent the health-care system itself. This region faced health system challenges such as health inequity and increased health-care costs, and the governments needed to build strategies that ensured accessibility to high-quality health care [54]. To pursue this, Pakistan conducted outreach research in the EMR and launched the government-led “lady health worker” program. They provided outreach services for minority populations, including maternal, newborn and child health services and health promotion referrals [55]. Community pharmacies are also considered ideal facilities for providing services such as education, detection and referral of patients [56]. A pharmacy-based CVD risk-screening program was implemented among high-risk CVD patients in the EMR [57]. In this respect, EMR governments have been attempting to take preventive measures at the community level to strengthen primary health-care provision.

In the WPR, the keywords “home” and “model” were prevalent. In Korea, community nursing services consisted of hospital-centered home health nursing and community-based visiting nurse services. Nurses delivered case management programs for patients suffering from chronic diseases [58] and these programs suggest that case management can improve patients’ outcomes [59]. In Japan, community outreach is a crucial component of the mental health-care model, which has been widely implemented and researched through assertive outreach [60]. In Australia, to improve access to health-care services, many service delivery models have been applied and showed to have efficacy in rural and remote areas, often being integrated with comprehensive primary health-care services [61].

In this study, the LDA method was adopted to detect various focus topics in outreach research based on the derived keywords. By categorizing the keywords, we derived the following five meaningful topic groups. The first group consisted of keywords related to “patient-centered care.” We found that the keyword “patient” reflected the highest frequency and centrality, with emphasis on it growing over time. This may be because health-care delivery has been transitioning over the past decade, with the WHO reporting that these changes were moving toward patient-centered health services—approaches that link patients directly with health professionals [6]. These new service delivery systems offer community health centers the opportunity to provide patient care more flexibly and allow the use of health outreach programs [62].

The second group comprised keywords related to “HIV care continuum.” In this study, the keyword “HIV” ranked high across periods and countries, and is among the most active outreach programs and services currently. “HIV care continuum” refers to a step-by-step process from HIV diagnosis through to treatment until viral load is suppressed to undetectable levels. The process includes diagnosis, link to medical care, ART, adherence to treatment regimen and viral load suppression to undetectable levels in the blood [63]. Outreach teams targeted this process to specific groups to reduce ongoing transmission and improve health outcomes and suggested universal testing and treatment strategies [64].

The third group comprised keywords associated with “services related to specific diseases.” There exist substantial health disparities among women worldwide, wherein minority women face high mortality rate and delays in receiving treatment [65]. Many countries have executed programs to increase cancer screening efforts and improve mental health among minority female groups through education and outreach activities, an endeavor that has proven successful in improving intervention effectiveness and clinical trial retention [65,66,67].

The fourth group is related to “community-based health-care services”. Community-based care is critical in providing continuous care to underserved populations. A sustainable outreach service can be consolidated with primary health care to form a well-organized aid that is responsive to community needs [68]. Many community-based outreach programs were developed to reduce disease-related disparities among underserved populations by identifying needs and barriers to care and attempting to increase access to health care. Meta-analysis of the effectiveness of preventive primary care outreach interventions showed that such programs aimed at older people were associated with a 17% reduction in mortality and a 23% increase in the likelihood of continuing to live in the community [7].

The fifth group is that of keywords linked to “research and education on health programs.” Given the importance of primary care, there are growing outreach intervention studies on its effectiveness and impact on health outcomes in primary care settings [7,69]. Education is considered an attribute of patient-centered care [67], and many outreach intervention studies have examined the effects of education and training on patients, community health workers and students who participate in outreach projects. Community outreach programs provide education to raise awareness about specific health matters, available services and the importance of various services [13,67]. Future outreach research needs to emphasize education as one of the key attributes of patient-centered care in primary care settings.

This study was restricted by a methodological limitation worth noting here. Specifically, we analyzed health-care-related outreach research trends by region according to the WHO regional criteria; therefore, the interpretation or application of the study outcomes must be done considering the confines of these criteria. Additionally, since we analyzed trends in the research by 10-intervals, there is a limit to grasping changes in a short time.

Moreover, there are limitations of the TNA method that is used to collect big data through a quantitative method and to identify a relationship network between keywords. In TNA, it is highly likely that researchers rely on their knowledge, experience and insights during analyzing data and grasping its meaning. In this study, therefore, it was possible to identify the important semantic contexts of analysis results through an inductive approach based on data, excluding the subjectivity of the researcher. In addition, the extracted text was collected only from the titles and abstracts of published articles, and keywords with low frequency and low centrality were excluded before analysis in this study. Therefore, generalization of these results should be carefully done through reasoning and evidence.

## 5. Conclusions

Using text network analysis, we were able to derive the implications of the importance of outreach research through a variety of approaches including period and country specific analysis as well as topic modeling. To our knowledge, this is the first study to identify the knowledge structure of outreach and trends in this topic of research. Our study revealed that research of outreach programs in health-care settings has focused on the five topic groups: patient-centered care, HIV care continuum, services related to a specific disease, community-based health care service, research and education of health program.

Until the present, the global outreach research has been mainly conducted with the providing services in community care programs to solve the health inequity by increasing access to health services. Although this fundamental perspective of outreach programs was maintained, our results newly identified that patient-centered care has been growing more crucial for all times and countries of the world by the examination of major trends in health-care related outreach research by time and region. In terms of target population and disease, there is awareness that some populations such as children and women are still target groups who need the services and outreach programs and services are becoming diversified with increased focus on chronic diseases such as breast and colon cancer as well as HIV/AIDS.

Our study revealed that the importance of outreach research is increasing in recognizing and diminishing health-care inequalities within the health-care system. The knowledge structure identified in this study can broaden a systematic understanding of outreach research. Health professionals including nurses and policymakers also gain an important insight into health-care planning to improve health inequality in community practice settings and can provide high quality health services through proper education and training. In future research, we propose to integrate the outreach programs and interventions into patient-centered care and to expand to diverse hard-to-reach populations and various chronic disease. Ultimately, these can make a positive contribution to the future direction in recognizing research, education, practice fields and diminishing health-care inequalities within the health-care system.

## Figures and Tables

**Table 1:** Top 30 keywords that emerged from the outreach research.

Rank	Keyword	Frequency	Keyword	Centrality Degree	Keyword	Betweenness Centrality
1	patient	21,744	patient	0.3852	patient	0.1365
2	care	14,901	health	0.3234	health	0.0932
3	health	14,675	program	0.3132	program	0.0757
4	service	13,328	service	0.3125	service	0.0700
5	program	12,675	care	0.2914	community	0.0564
6	community	10,584	community	0.2616	care	0.0484
7	intervention	10,110	intervention	0.2144	treatment	0.0209
8	treatment	7617	treatment	0.1991	intervention	0.0202
9	woman	7261	education	0.1577	HIV	0.0183
10	risk	5749	woman	0.1504	risk	0.0174
11	HIV	5696	risk	0.1468	woman	0.0168
12	child	5539	practice	0.1439	child	0.0139
13	clinic	5499	clinic	0.1417	clinic	0.0129
14	practice	5414	access	0.1374	screening	0.0127
15	education	5221	HIV	0.1352	practice	0.0126
16	research	5174	child	0.1352	research	0.0116
17	need	5155	outreach	0.1308	education	0.0104
18	hospital	4739	research	0.1279	access	0.0102
19	person	4720	need	0.1265	cost	0.0100
20	screening	4602	provider	0.1250	knowledge	0.0097
21	model	4507	hospital	0.1228	drug	0.0088
22	access	4437	screening	0.1221	area	0.0088
23	outreach	4312	person	0.1206	system	0.0085
24	strategy	4132	family	0.1163	disease	0.0084
25	provider	3935	support	0.1156	country	0.0080
26	information	3922	knowledge	0.1112	hospital	0.0078
27	center	3715	information	0.1097	testing	0.0077
28	team	3710	management	0.1083	model	0.0075
29	support	3661	team	0.1054	worker	0.0070
30	area	3525	training	0.1054	activity	0.0070

**Table 2:** Top 30 Keywords of Degree Centrality over Time.

Rank of Degree Centrality	≤1990	1990s	2000s	2010s
1	program	program	service	patient
2	service	health	health	health
3	health	service	program	care
4	community	community	patient	service
5	care	care	community	program
6	child	patient	care	community
7	area	child	intervention	intervention
8	hospital	treatment	treatment	treatment
9	education	woman	child	risk
10	health care	intervention	woman	education
11	clinic	clinic	practice	HIV
12	family	HIV	education	clinic
13	patient	education	risk	practice
14	center	practice	need	screening
15	treatment	health care	research	outreach
16	problem	prevention	outreach	access
17	adolescent	access	HIV	research
18	need	risk	clinic	provider
19	cost	need	management	woman
20	immunization	hospital	access	need
21	prevention	information	health care	support
22	staff	center	Team	child
23	woman	knowledge	information	hospital
24	Mental Health	outreach	staff	person
25	cancer	system	screening	family
26	development	person	cancer	knowledge
27	person	family	hospital	training
28	physician	drug	system	team
29	research	nurse	development	information
30	teen	project	cost	management

**Table 3:** Top 30 Keywords of Degree Centrality each region.

Degree Centrality	AFRO	AMR	SEARO	EURO	EMRO	WPRO
1	health	patient	health	patient	health	service
2	service	program	service	service	service	health
3	community	health	program	care	community	patient
4	HIV	care	community	health	patient	care
5	program	service	patient	intervention	care	community
6	patient	community	care	community	program	program
7	care	intervention	HIV	treatment	awareness	intervention
8	child	treatment	woman	program	disease	clinic
9	facility	education	India	team	family	treatment
10	treatment	woman	treatment	practice	access	hospital
11	woman	provider	intervention	hospital	prevention	child
12	intervention	risk	hospital	clinic	delivery	person
13	training	research	awareness	person	hospital	access
14	clinic	screening	prevention	HIV	intervention	model
15	prevention	outreach	access	risk	treatment	specialist
16	support	practice	center	nurse	HCV	staff
17	access	child	risk	need	coverage	management
18	district	HIV	child	support	healthcare	support
19	hospital	clinic	area	management	pharmacy	practice
20	team	need	delivery	child	system	family
21	delivery	access	facility	staff	woman	risk
22	Community Health Worker	family	health care	outreach	Pakistan	disease
23	disease	information	cancer	Mental Health	cancer	education
24	education	cancer	clinic	education	center	nurse
25	strategy	knowledge	education	training	health care	HIV
26	worker	student	practice	cost	outreach	knowledge
27	cost	center	government	specialist	referral	strategy
28	need	health care	knowledge	testing	risk	team
29	immunization	barrier	development	Primary Care	HIV	delivery
30	TB	prevention	cost	family	Hypertension	home

**Table 4:** Topic modeling of outreach research.

Topic Groups (n, %)	Keywords (Weight)
Topic 1 Patient-centered care (3170, 25%)	patient (0.084), care (0.038), hospital (0.018), intervention (0.018), practice (0.012), clinic (0.012), service (0.012), team (0.011), management (0.009), visit (0.009)
Topic 2 HIV care continuum (1828, 14%)	HIV (0.043), testing (0.021), screening (0.02), risk (0.016), treatment (0.016), intervention (0.014), infection (0.014), drug (0.013), man (0.012), HCV (0.012)
Topic 3 Services related to a specific disease (2130, 17%)	woman (0.023), treatment (0.02), cancer (0.019), service (0.014), health (0.014), screening (0.012), risk (0.011), Mental Health (0.011), care (0.011), intervention (0.01)
Topic 4 Community-based health care service (2428, 19%)	service (0.037), child (0.029), health (0.026), care (0.017), woman (0.015), program (0.015), community (0.017), intervention (0.01), access (0.009), need (0.008)
Topic 5 Research and education of health program (3331, 26%)	health (0.028), community (0.025), program (0.024), research (0.015), student (0.015), education (0.012), training (0.01), information (0.009), project (0.009), activity (0.008)

## Data Availability

Data available in a publicly accessible repository that does not issue DOIs.

## Supplementary Materials

The following are available online at https://www.mdpi.com/article/10.3390/ijerph18179309/s1, Table S1: Search strategy in the databases, Table S2: The excel form of extraction from included studies, Figure S1: The number of outreach articles from 1975–2020.
