# Supplementary material for: Identifying the Knowledge Structure and Trends of Outreach in Public Health Care: A Text Network Analysis and Topic Modeling
Source: Int J Environ Res Public Health. 2021 Sep 3;18(17):9309. doi: 10.3390/ijerph18179309 (PMC8431096; doi:10.3390/ijerph18179309)
Supplement: Supplementary file 1 [file ijerph-18-09309-s001.zip › ijerph-1336771-supplementary.pdf]

Table S1. Search strategy in the databases

		Search query	Results
PubMed	3	"outreach"[Title/Abstract] AND ("care"[Title/Abstract] OR "health"[Title/Abstract] OR "healthcare"[Title/Abstract]) AND ("humans"[MeSH Terms] AND "english"[Language])	**7,643**
	2	"care"[Title/Abstract] OR "health"[Title/Abstract] OR "healthcare"[Title/Abstract]	2,939,814
	1	"outreach"[Title/Abstract]	14,233
Embase	#7	#1 AND #5 AND [humans]/lim AND [english]	**12,823**
	#6	#1 AND #5	14,213
	#5	#2 OR #3 OR #4	3,850,261
	#4	healthcare:ab,ti	386,551
	#3	health:ab,ti	2,484,104
	#2	care:ab,ti	1,984,974
	#1	outreach:ab,ti	19,768
CINAHL	S6	(S2 OR S3 OR S4) AND (S1) AND limited [humans] and [english]	3,334
	S5	S2 OR S3 OR S4	1,564,022
	S4	TI healthcare OR AB healthcare	326,263
	S3	TI health OR AB health	996,270
	S2	TI care OR AB care	868,074
	S1	TI outreach OR AB outreach	9,020

Table S2. The excel form of extraction from included studies

Author	Year	Title	Journal	Volume	Issue	Date	Type of Article	ISSN	DOI	Keywords	Abstract	URL	Author Address	Country	Region_ WHO

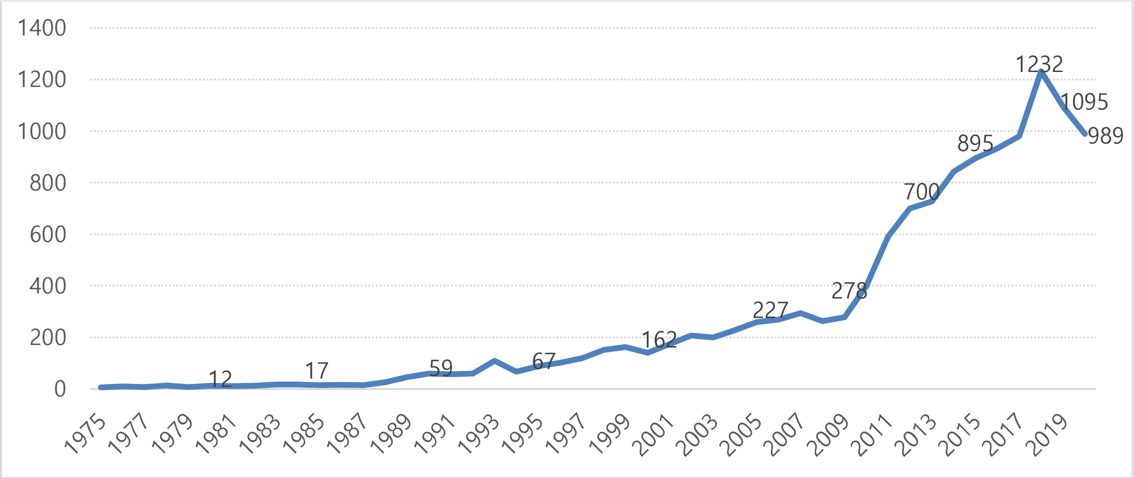

Figure S1. The number of outreach articles from 1975–2020.
